# Exploring the Exopolysaccharide Production Potential of Bacterial Strains Isolated from Tunisian Blue Crab *Portunus segnis* Microbiota

**DOI:** 10.3390/molecules29040774

**Published:** 2024-02-07

**Authors:** Mariem Migaou, Sabrina Macé, Hana Maalej, Laetitia Marchand, Sandrine Bonnetot, Cyril Noël, Corinne Sinquin, Marc Jérôme, Agata Zykwinska, Sylvia Colliec-Jouault, Raoui Mounir Maaroufi, Christine Delbarre-Ladrat

**Affiliations:** 1 Laboratory of Genetics, Biodiversity & Valorisation of Bioresources, Higher Institute of Biotechnology of Monastir, University of Monastir, Ave Tahar Haddad, BP74, Monastir 5000, Tunisia; 2 Ifremer, MASAE Microbiologie Aliment Santé Environnement, F-44000 Nantes, Francecorinne.sinquin@ifremer.fr (C.S.);; 3 Laboratory of Biodiversity and Valorization of Arid Areas Bioresources, Faculty of Sciences, University of Gabès, Erriadh, Zrig, Gabès 6072, Tunisia; 4 Ifremer, IRSI, SeBiMER Service de Bioinformatique de l’Ifremer, F-29280 Plouzané, France

**Keywords:** *Portunus segnis*, 16S rDNA sequencing, microbiota, bacteria, exopolysaccharide

## Abstract

The blue crab (BC) *Portunus segnis* is considered an invasive species colonizing Tunisian coasts since 2014. This work aims to explore its associated bacteria potential to produce anionic exopolysaccharides (EPSs) in order to open up new ways of valorization. In this study, different BC samples were collected from the coastal area of Sfax, Tunisia. First, bacterial DNA was extracted from seven different fractions (flesh, gills, viscera, carapace scraping water, and three wastewaters from the production plant) and then sequenced using the metabarcoding approach targeting the V3-V4 region of the 16S rDNA to describe their microbiota composition. Metabarcoding data showed that the dominant bacterial genera were mainly *Psychrobacter*, *Vagococcus*, and *Vibrio*. In parallel, plate counting assays were performed on different culture media, and about 250 bacterial strains were isolated and identified by sequencing the 16S rDNA. EPS production by this new bacterial diversity was assessed to identify new compounds of biotechnological interest. The identification of the bacterial strains in the collection confirmed the dominance of *Psychrobacter* spp. strains. Among them, 43 were identified as EPS producers, as revealed by Stains-all dye in agarose gel electrophoresis. A *Buttiauxella* strain produced an EPS rich in both neutral sugars including rare sugars such as rhamnose and fucose and uronic acids. This original composition allows us to assume its potential for biotechnological applications and, more particularly, for developing innovative therapeutics. This study highlights bacterial strains associated with BC; they are a new untapped source for discovering innovative bioactive compounds for health and cosmetic applications, such as anionic EPS.

## 1. Introduction

The Mediterranean Sea, one of the most complex marine ecosystems and considered a marine biodiversity hotspot, is inhabited by a rich and diverse biota, among which crustaceans constitute the second most represented taxon of non-native species [1]. The blue swimming crab (BC), *Portunus segnis*, was the first Lessepsian migrant crustacean that reached, in 1898, the Mediterranean Sea shortly after the opening of the Suez Canal [2,3]. Its occurrence along the Tunisian coast was first recorded in 2014 in the south-eastern Tunisian Gulf of Gabes. Since August 2015, there has been a tremendous expansion of this crustacean which has led to severe ecological and socioeconomic impacts by displacing native species and changing the community structure and food webs [4,5,6,7]. To overcome this situation, studies were funded to promote its exploitation and valorization, thus aiming at turning this threat into an opportunity [8]. Nowadays, BC *Portunus segnis* represents a precious fishery resource as a shellfish food product, and its processing co-products have become a valuable bioresource investigated to develop high-value biochemical compounds such as chitin, chitosan and chitooligosaccharides [9,10], carotenoids [11], and digestive enzymes [12,13]. However, so far, to the best of our knowledge, there is no study undertaken to explore BC microbial communities, although this kind of bacteria are a promising source for the discovery of molecules of interest.

New bioactive compounds produced by marine bacteria with a large variety of biological functions have been described and they present great potential for both biotechnological and industrial applications [14,15,16]. Particularly, some marine bacterial species isolated from various sample sources (clams, oysters, shrimps, polychaete annelids, and hydrothermal fluid…) and environments (deep-sea hydrothermal vents, Antarctic Sea ice, and Polynesian microbial mats…) have been reported to produce anionic exopolysaccharides (EPSs) composed of different sugars that can also present non-sugar substituents [17,18]. Marine bacterial EPSs are carbohydrate polymers presenting complex structures endowed with unique biological activities and functions; they are attractive targets for pharmaceutical and biomedical fields, including antifreeze, antioxidant, anticancer, anti-inflammation, immune, and antibacterial activities [19]. In addition, marine EPSs have versatile applications in bioremediation, wastewater treatment, heavy metal treatment, and marine oil pollution [20].

Nowadays, molecular taxonomic tools can accurately determine bacterial diversity, obviating the need for laboratory strain cultivation [21]. Among these tools, next-generation sequencing (NGS) explores global genetic biodiversity and can uncover new microorganisms [22]. In this work, *P. segnis* microbiota was determined using metabarcoding targeting the 16S rRNA gene. In parallel, the characterization and identification of isolated strains were carried out, along with the screening of anionic EPSs. To the best of our knowledge, this work is the first one studying the microbiota of this BC species collected from Sfax Tunisian coasts, and its exploration is crucial to decrypt its bacterial diversity and find microorganisms of biotechnological interest. We focused our study on bacterial anionic EPSs and carried out the screening using agarose gel electrophoresis.

## 2. Results

### 2.1. Enumeration of the Different Bacterial Groups

**Figure 1:**
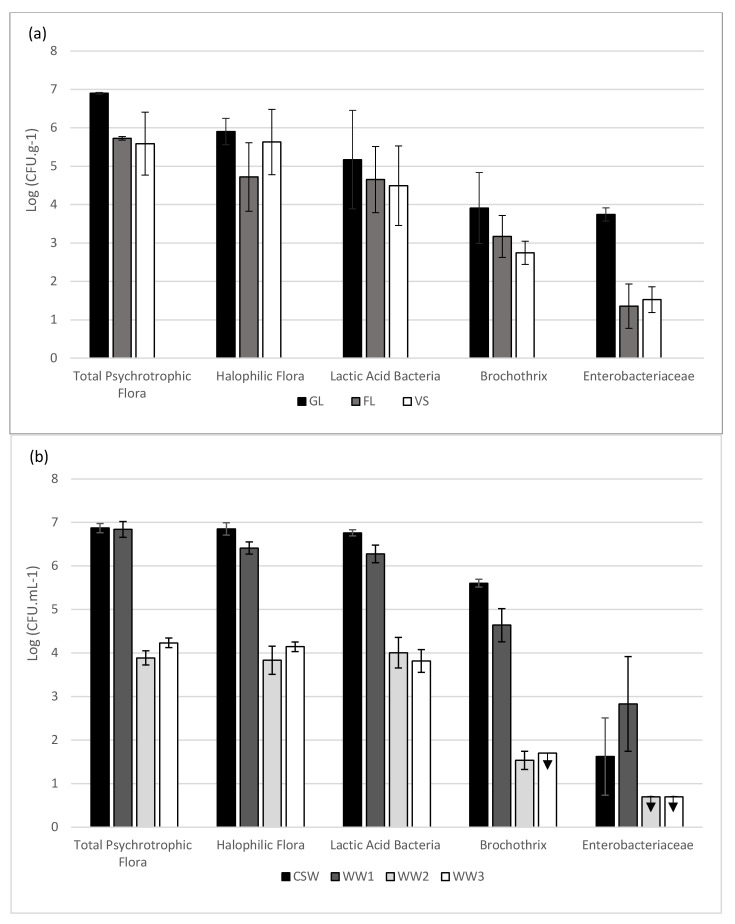
Bacteria cell numbers of the 3 different samples from blue crab (**a**), and carapace scraping water and the 3 washing waters used in the industrial production process (**b**). GL: gills, FL: flesh, VS: viscera, and CSW: carapace scraping water. WW: wastewater from different industrial processing steps; black arrow symbol (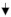) represents the results under the limit of plate counting quantification for 3 replicates. Results are expressed as mean and standard deviation of 3 biological replicates.

In the seven different fractions including crab-related samples like flesh (FL), gills (GL), viscera (VS), carapace scraping water (CSW), and industry wastewaters (WW1, WW2, and WW3), cultivable bacteria were enumerated based on selected appropriate culture media (Figure 1).

Variable amounts of bacterial cells were calculated in the different crab fractions (Figure 1a). For all crab samples, the main strains found were psychrotrophic flora with concentrations between 7 Log (CFU g^−1^) in GL and about 5.5 Log (CFU g^−1^) in VS. For GL, halophilic flora and lactic acid bacteria (LAB) reached, respectively, 5.9 Log (CFU g^−1^) and 5.2 Log (CFU g^−1^). FL bacterial concentrations reached a level close to 5.5 for psychrotrophic flora and 4.5 Log (CFU g^−1^) in both halophilic flora and LAB. Finally, the VS bacterial concentration reached values of approximately 5.5 Log (CFU g^−1^) for halophilic flora and 4.5 Log (CFU g^−1^) for LAB. *Brochothrix* spp. concentrations were quite close for all of the types of samples, reaching about 4 Log (CFU g^−1^) for GL and about 3 Log (CFU g^−1^) for FL and VS. Concerning the Enterobacteriaceae family, GL was the main bacteria-containing fraction, with a reached concentration of approximately 4 Log (CFU g^−1^) followed, respectively, by VS and FL with, respectively, 1.5 and 1.3 Log (CFU g^−1^) concentrations.

For wastewater (WW) and carapace scraping water (CSW) (Figure 1b), the dominating bacteria were the same as for crab fractions. Psychrotrophic flora, halophilic flora, and LAB exhibited a similar distribution pattern in CSW, WW1, and WW2, with the highest concentration close to 7 Log (CFU mL^−1^) in CSW and WW1, while WW2 and WW3 reached about 4 Log (CFU mL^−1^). Halophilic flora and LAB presented almost the same concentration as psychrotrophic flora for each sample, suggesting that those two bacterial groups might have a dominant part in the ecosystem. *Brochothrix* concentration was about 5.6 Log CFU mL^−1^ for CSW, 5 Log CFU mL^−1^ for WW1, 1.5 Log CFU mL^−1^ for WW2, and under the limit of detection (<1.7 CFU mL^−1^) for WW3. The Enterobacteriaceae family was the group present in the lowest amount in the WW. The WW1 concentration was the highest with nearly 3 Log (CFU mL^−1^), and lower levels were registered for WW2 and WW3, which were below the detection limit (< 0.7 Log CFU mL^−1^). The Enterobacteriaceae concentration was the lowest present in crab WW. WW1 was the highest level, which was nearly 3 Log CFU mL^−1^, followed by CSW with a concentration of about 1.6 Log CFU mL^−1^, while the levels for WW2 and WW3 were under the limit of detection.

### 2.2. Bacterial Identification by 16S rRNA Gene Sequencing

The 247 collected isolates were identified by 16S rRNA gene sequencing (Supplemental Data I). The results showed a distribution of 27 different genera (Table 1).

Only two bacterial classes were represented in the collection (Supplemental Data I): Bacilli (phylum Bacillota; *n* = 80) and Gammaproteobacteria (phylum Pseudomonadota, *n* = 167). Two orders of Bacilli with three families in each of them were found: Listeriaceae, Planococcaceae, and Staphylococcaceae in 28 Bacillales; Carnobacteriaceae, Enterococcaceae, and Streptococcaceae in 52 Lactobacillales. Gammaproteobacteria were more diverse since they were distributed into six families: 6 Aeromonadales (Aeromonadaceae), 6 Alteromonadales (Shewanellaceae), 26 Enterobacterales (Enterobacteriaceae, Morganellaceae, Yersinaceae), 96 Moraxellales (all Moraxellaceae, including *Psychrobacter* genus), 1 Pseudomonadale (Pseudomonadaceae), and 32 Vibrionales (all Vibrionaceae family and *Vibrio* genus) were identified. Therefore, a core community of Gammaproteobacteria was described for this bacterial strain collection of crab-associated microbiota.

More than 50 different species (*n* = 51) were identified in all samples. The 247 bacterial strains represented 27 distinct genera, each including between 1 and 15 identified species, with the highest number for *Psychrobacter* genus. However, identification at the species level was not always possible since no species could be assigned to 55 strains in 14 different genera (sp. identifications). The most abundant genus was *Psychrobacter* with 95 isolates, followed by *Vibrio* (*n* = 32), *Lactococcus* (*n* = 24), *Vagococcus* (*n* = 13), and *Serratia* (*n* = 11). There were common strains shared between all samples, such as *Lactococcus garviae* (*n* = 24), *Vibrio alginolyticus* (*n* = 22), and *Psychrobacter celer* (*n* = 21). Three genera (*Vibrio*, *Psychrobacter*, and *Lactococcus*) were distributed in all crab fractions and water samples, while *Vagococcus*, *Enterococcus*, *Jeotgalibaca* and *Jeotgalicoccus* genera were mainly found in carapace scraping water (CSW) and wastewaters (WW1, WW2, and WW3). Strains of *Macrococcus* genus were also present in BC, particularly in the flesh part (FL).

Surprisingly, the third BC wastewater (WW3) displayed the highest number of distinct genera and species (14 different); in addition, the highest number of isolates was also obtained from this sample (*n* = 45). Viscera (VS) also exhibited a large diversity, with 14 different genera, while flesh (FL) was the least rich sample (9 distinct genera). Also, through analyzing the results of 16S according to the isolation media, a large number of isolated strains on Elliker were identified as *Psychrobacter* sp., representing 50% of the total number of isolated colonies which were also observed on Zobell and LH media. These results may be explained by the significant existence of *Psychrobacter* bacterial strains in the marine environment [23]. Moreover, most *Psychrobacter* sp. grow more at an optimum temperature between 20 and 30 °C and with the presence of ≥10% (*w*/*v*) NaCl. *Psychrobacter sanguinis* is the least halotolerant, for which the upper NaCl level in the growth media is 2% [24].

### 2.3. Bacterial Diversity Using Metabarcoding Analysis

The 16S rRNA amplicon sequencing of the 21 samples resulted in 3,121,586 reads, of which 25% were successfully merged. During the analysis of metabarcoding data by SAMBA, 520 ASVs (amplicon sequence variants) were assigned. For the diversity analysis, VS, FL, and GL as well as CSW were grouped as BC samples, while WW1, WW2, and WW3 were grouped as wastewater samples. Repartition of the ASVs among these samples showed that 265 and 112 ASVs were specific for the WW and BC matrix samples (FL, GL, VS, and CSW), respectively, with 143 shared common ASVs. The alpha diversity based on the Shannon diversity index for the BC samples presented lower values of, respectively, 2.5 (FL), 2.55 (GL), 2.72 (VS), and 2.29 (CSW). Water samples collected from the factory process showed slightly higher index values of 3.43 (WW1), 3.37 (WW2), and 2.9 (WW3).

**Figure 2:**
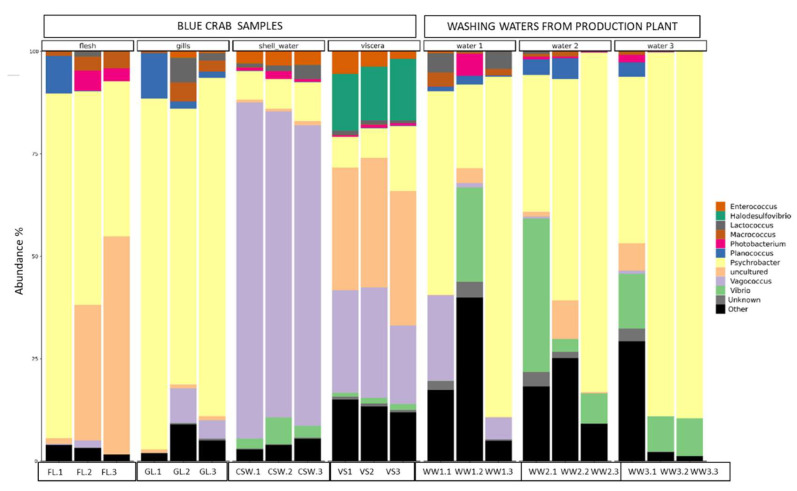
Relative abundance of the ten dominant bacterial genera associated with the microbiota of *Portunus segnis* and the microbiota of BC wastewaters recovered from different industrial processing steps.

The results of the amplicon sequencing illustrated how each sample fraction had its own diversity (Figure 2). For each type of sample, the triplicate was mostly homogeneous: they contained the same bacterial genera except for the first replica of flesh and gills, as well as for the second replicate of WW1. *Psychrobacter* sp. was the main genus in all samples (between 30 and 80% of relative abundance) except for CSW and VS, which both were enriched with *Vagococcus* sp. (30–70% of relative abundance). Uncultured bacteria also displayed an important proportion in VS samples (30%) as well as in two flesh samples (30–50%). In some WW samples, *Vibrio* can represent 10–30%. Also, WW1 was found to be relatively heterogeneous with *Vagococcus* sp., present both in first and third replicates, while *Vibrio* sp. was present in the second replicate, similarly to WW2 and WW3.

In addition to the *Psychrobacter*, *Vagococcus*, and *Vibrio* main genera, other species, such as *Planococcus*, *Enterococcus*, *Photobacterium*, and *Lactococcus* sp., were also identified in the samples. *Halodesulfovibrio* sp. was specifically identified in the viscera. This resulted in a decrease in *Planococcus* ASV relative abundance in both FL and GL, and an increase in the uncultured group and *Photobacterium* in FL and *Vagococcus* sp. in GL.

### 2.4. EPS-Producing Strains

Two hundred and forty seven (247) strains isolated from the crab microbiota were screened for the production of EPSs; they were grown in 1 mL of Zobell–glucose medium and incubated for 48 h at 20 °C. After concentration, the supernatant was analyzed by agarose gel electrophoresis using Stains-all, which stains anionic molecules.

In total, 43 bands corresponding to anionic molecules were observed on agarose gel, suggesting that 43 among the 247 bacterial strains could produce EPSs under the chosen screening conditions (Supplemental Data I). A total of 24 strains were isolated on Zobell medium, 7 on PCA, 5 on BHI-S, 4 on LH, and 3 on Elliker.

The majority of the 43 EPS-producing bacteria were Gammaproteobacteria (*n* = 43); no bacterial strain belonging to the Bacilli family was shown to produce an anionic compound, nor the sole Pseudomonadaceae strain. Positive strain diversity was similar to the strain collection: 3 Aeromonadaceae (genus *Aeromonas*), 9 Enterobacteriaceae (genera *Buttiauxella*, *Enterobacter*, *Escherichia*, *Morganella*, *Proteus*, and *Raoultella*,), 14 Moraxellaceae (genus *Psychrobacter*), 4 Shewanellaceae (genus *Shewanella*), and 13 Vibrionaceae (genus *Vibrio*). Eight EPS-producing strains were isolated from viscera, seven from gills and the third wastewater WW3, and six from flesh, WW1, and WW2. Among these 43 strains, 12 were selected for further investigation. Their identification was confirmed by 16S rRNA gene sequencing (Table 2). The selection of the most interesting presumed anionic EPS-producing strains was based on migration pattern in the electrophoresis gel, diversity of the genera, and origin, while avoiding the recognized human–pathogenic genus.

**Figure 3:**
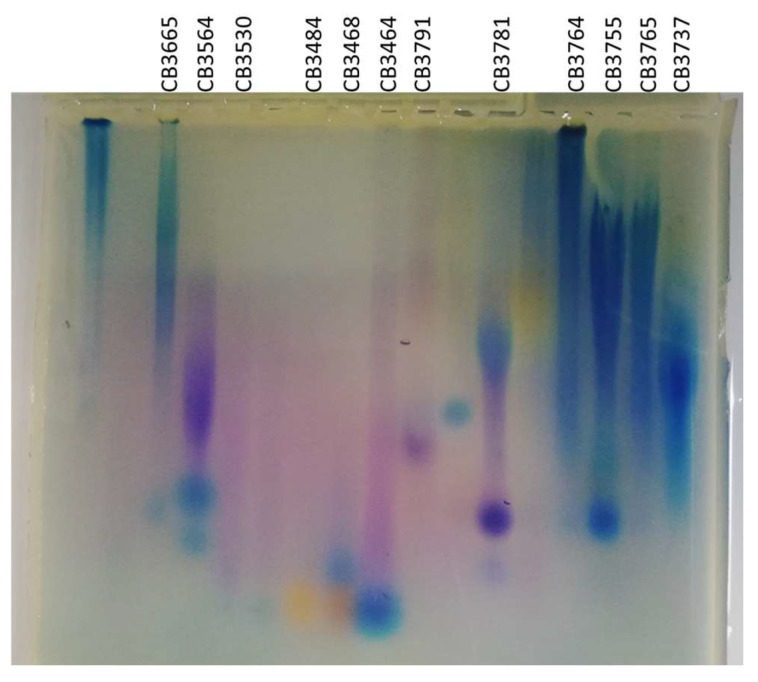
Electrophoresis gel showing EPSs produced by the 12 selected isolates. The analysis was performed by agarose gel electrophoresis using Stains-all as dye; no marker is available for unknown polysaccharides as they migrate both upon the Mw and the charge.

Selected strains were then grown in larger volumes (600 mL of Zobell–glucose medium in baffled flasks) in order to confirm the EPS production. Produced EPSs were first analyzed by gel electrophoresis then characterized by their amount produced, their content in monosaccharides, and their molecular weight (Figure 3 and Table 2).

Staining of the produced EPSs after migration in agarose gel suggested that these polymers have an anionic charge that could be due to the presence of acidic sugars or substituents such as sulphate or organic acid groups (e.g., lactate or pyruvate). Different patterns were observed with distinct colors, as described by Andrade et al. [25], suggesting different EPS types: a blue broad smear is characteristic of acidic EPS for the strains CB3665, CB3764, CB3755, CB3765, and CB3737; purple/pink spot or smear is often obtained for sulphated EPS for CB3564 with a deep color and for CB3530, CB3484, CB3468, CB3464, and CB3791 with a light purple band; and the presence of both blue and pink spots is characteristic for CB3781.

The production and molecular weight of EPSs produced by different strains are presented in Table 2. The obtained concentration in the culture broth varied from 10.5 mg·L^−1^ for CB3530 (a *Psychrobacter* strain close to *P. marincola* and *submarinus*) to 127.8 mg·L^−1^ for CB3765 (*V. alginolyticus*). This concentration is below the EPS production generally encountered for marine EPSs, which is around 1 g·L^−1^ in a bioreactor; however, the production in shake flasks is usually less efficient than in a bioreactor [26]. For strains displaying low production of EPSs, we expect that a subsequent optimization of culture conditions would allow for a much better harvest. The weight-average molecular weight (Mw) ranged from 74 × 10^5^ g/mol for CB3564 (*Psychrobacter* sp.) to 81 × 10^6^ g/mol for CB3765 (*V. alginolyticus*), exceeding the weight observed usually for bacterial EPSs [20]. Most EPS molecular weights exceeded the fractionation domain of the column; this could be due to aggregations of polysaccharidic chains in the solution.

**Figure 4:**
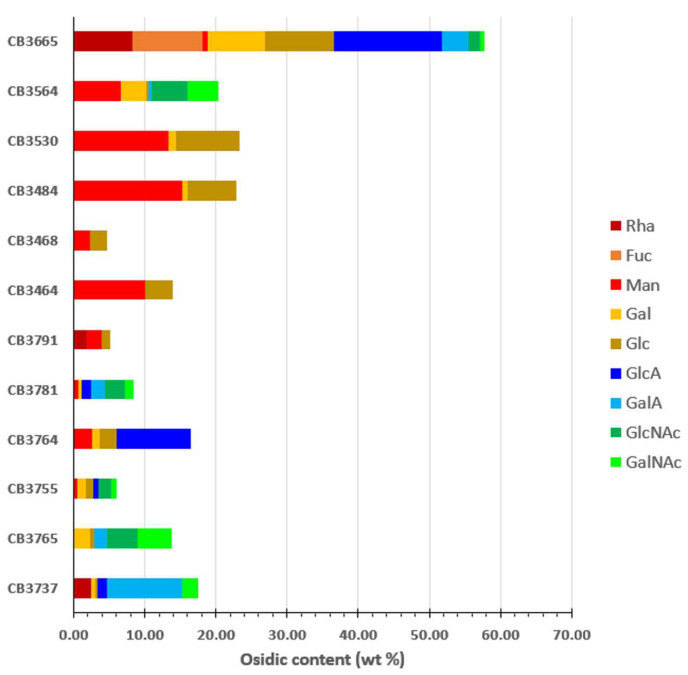
Monosaccharide composition (wt%) of the selected EPSs produced by indicated bacterial strains. Monosaccharides: fucose (Fuc); galactose (Gal); galacturonic acid (GalA); glucose (Glc); glucuronic acid (GlcA); mannose (Man); N-Acetyl-glucosamine (GlcNAc); N-Acetyl-galactosamine (GalNAc); rhamnose (Rha).

The monosaccharide composition of these EPSs showed important diversity (Figure 4). EPSs produced by *Buttiauxella* sp. (CB3665) presented the highest content of total sugars and also the most complex osidic composition, containing both neutral sugars, including rhamnose (Rha), fucose (Fuc), galactose (Gal), and glucose (Glc), and uronic acids, such as glucuronic acid (GlcA) and galacturonic acid (GalA). Only neutral sugars, mainly mannose (Man) and Glc, were found in EPSs produced by four *Psychrobacter* sp. strains (CB3464, CB3468, CB3484, and CB3530), while *Psychrobacter* strain CB3564 contained, in addition, acetylated hexosamines such as N-acetyl-galactosamine (GalNAc) and N-acetyl-glucosamine (GlcNAc). The elemental analysis performed on these EPSs showed a very low content of sulfur, suggesting that the observed acidic nature in agarose gel could be due to the presence of other acidic groups such as pyruvate or acetate. In addition to EPSs from *Buttiauxella* sp, two other EPSs produced by *S. algae* (CB3764) and *V. furnissii* (CB3737) were particularly rich in uronic acids. EPSs produced by *V. alginolyticus* (CB3781 and CB3765) were rich in acetylated hexosamines and contained some uronic acids, the composition usually described for EPSs secreted by *V. alginolyticus* strains [27,28].

## 3. Discussion

In this study, we explored the microbiota of Tunisian blue crab (BC), *Portunus segnis*, and isolated 247 bacterial strains, which were identified and screened for anionic EPS production. Bacterial identification using the 16S rRNA gene sequencing of strains isolated from different media (Elliker, Zobell, LH, PCA, and BHI-S) showed that the dominant genera were mainly *Psychrobacter*, *Vibrio*, and the lactic acid bacteria *Lactococcus* and *Vagococcus*. This is consistent with the results of the metabarcoding analysis, which showed that the dominant genera were *Psychrobacter*, then *Vagococcus*, uncultured bacteria, and *Vibrio*. Two other marine genera, i.e., *Photobacterium* and *Halodesulfovibrio*, were detected by NGS sequencing but not found by 16 S identification after cultivation of the isolated strains.

Specific microbiotas have been described for crab species [29,30,31,32]; these differences could be due to the geographic origin of crabs rather than the crab species itself. Some bacterial genera are noticed to be common between studies. For instance, since 1975, several investigators have shown the presence of *Vibrio* spp. associated with tissues of blue crabs collected in temperate waters [33,34]. This genus was found in our study as the second prevalent genus and was demonstrated as the most abundant genus in blue crabs *Callinectes sapidus* [35]. *Vibrio* spp. were detected also in the core intestinal microbiota of the mud crab *Scylla paramamosain* from the coast of southern China [32]. *Psychrobacter* spp. was identified in our study as the first dominant species; it was also identified as one of the most prevalent genera for other crabs, e.g., *Callinectes sapidus* [35] and *Chionoecetes* sp. collected at a seafood shop at Jukbyeon Harbor, South Korea [36], as well as in the fish *Salmo salar* [37] and other shellfish such as *Parapenaeus longirostris* shrimp [38]. Additionally, other genera such as *Photobacterium* and *Shewanella* recovered in the present study were also present in horseshoe crabs *Tachypleus gigas*, *Carcinoscorpius rotundicauda* collected from Balok Beach, Malaysia [39], and mud crabs *Scylla paramamosain* collected from Shantou, China [40].

Some of the bacterial strains isolated from the Tunisian BC samples, i.e., *Pseudomonas* spp., lactic acid bacteria, and Enterobacteriaceae, have been described in other studies to proliferate during the cold storage of fresh and cooked crabs, and could be involved in the spoilage [41,42,43]. In our study, fortunately, only a few potentially pathogenic bacterial strains of minor concern for human beings were present. Only one species of *Vibrio parahaemolyticus* was isolated and identified among the 247 strains This species has also been found in the blue swimming crab *P. pelagicus* [44]. In contrast, human pathogens such as *V. cholerae*, *V. parahaemolyticus*, and *V. vulnificus* have been identified in *C. sapidus* gills, viscera, flesh, and hemolymph [31].

Bacteria associated with marine animals have been described to have a symbiotic relationship with their hosts. The compounds produced by these marine bacteria can have an important physiological role for the crab or can be necessary for enhancing bacterial cell-to-crab interaction. Notably, EPS is a component of the biofilm extracellular matrix and allows bacterial cells to grow as a biofilm adhered to a surface, such as a crab surface.

In our study, the screening of EPS-producing strains was carried out to search specifically for anionic EPSs. Indeed, anionic EPSs can mimic glycosaminoglycans (GAGs), linear polysaccharides present on the cell surface and in the extracellular matrix of animal tissues, where they play a major role in both physiological and pathological processes [45,46]. They interact with a wide variety of proteins, such as cytokines, chemokines, or growth factors, and therefore regulate multiple cellular responses (e.g., inflammation and wound-healing processes). Consequently, GAGs are also therapeutically used in the biomedical field such as for tissue repair, cancer and metastasis inhibition, and Alzheimer’s disease treatment [47,48,49,50]. However, their structure heterogeneity and the complexity of their extraction and purification protocol, as well as the risk of contamination by viruses and non-conventional agents, can cause health issues and encourage scientific researchers to think about mimetic GAGs from other alternative sources [49,51,52].

In our work, we showed that less than 20% of the bacteria isolated from BC produced anionic EPSs. They all belonged to Gammaproteobacteria. In the blue crab samples, the three main genera producing anionic EPSs were *Vibrio*, *Shewanella*, and *Buttiauxella*. *Vibrio* sp. are well known for the production of EPSs exhibiting structural and biological similarities with GAGs [52,53]. *V. diabolicus * [54], *V. furnissii* VB0S3 [55], *V. harveyi* VB23 [56], *V. neocaledonicus* [57], *V. fischeri* [58], *V. parahaemolyticus* [59], and *Vibrio* sp. QY101 [60] have been described to secrete anionic EPSs; for some of them, diverse biological activities and biotechnological potential applications were identified, including antioxidant and antibacterial activities, and potential applications in human health and aquaculture. The chemical composition of several EPSs from *Vibrio* strains has been determined. This composition has been described to be strain-dependent and mainly composed of uronic acids and N-acetyl-hexosamines. Some of these EPSs were also decorated with amino acids, a property which was only detected by fine structural analysis [27,28,61]. The composition observed in our study is consistent with others described in the literature for the same genus, confirming that the EPS composition can be linked to the bacterial phylogenetic position [52,53]. *Alteromonas* and *Pseudoalteromonas* spp. were also described to produce anionic EPSs, but neither of these two genera were found in this study. Some studies on EPSs from *Shewanella* species were also conducted, such as *S. oneidensis* MR-1 [62] and *S. frigidimarina* W32–2 [63]. There is still little information reported regarding EPSs from *Psychrobacter* spp.; it would be worth studying further those identified in our work which were rich in neutral sugars. Their potential substitution with organic acids, such as pyruvate and acetate, could explain their migration and staining by Stains-all in agarose gel electrophoresis and their potential anionic nature. EPSs from *Psychrobacter* strains isolated from a bivalve *Ruditapes philippinarum*, from Antarctic ice samples and brine wastewater collected in a fish canning plant, were found to be composed of neutral or anionic sugars and to have interesting biological functions, such as antioxidant activity, macrophages stimulation, and initial immune response regulation, as well as physicochemical properties such as flocculation and discoloration, and can be used in high salinity wastewater treatment [64,65,66]. We also discovered a *Buttiauxella* sp. strain producing an interesting EPS rich in neutral sugars, including rare sugars such as Rha and Fuc, and uronic acids. To the best of our knowledge, this is the first time that an anionic EPS was reported in a *Buttiauxella* strain.

## 4. Materials and Methods

### 4.1. Blue Crab Sampling and Handling

**Figure 5:**
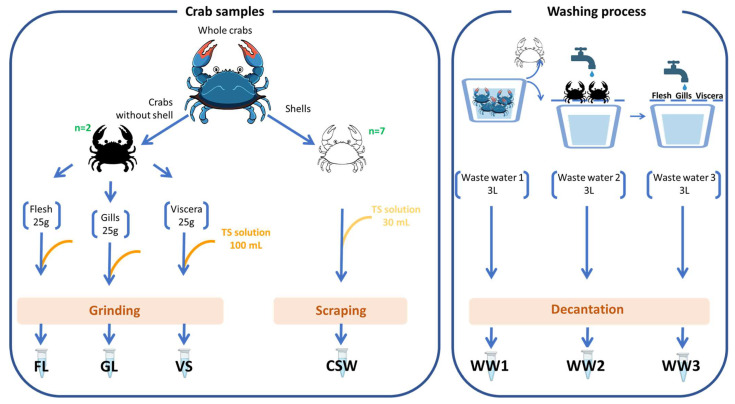
Sample preparation from blue crabs. Each sample and wastewater were prepared in triplicate. Image credits: blue crab: image of brgfx from Freepik; tube, tap water, and tanks from BioRender.com.

Sampling was carried out in 2021 within the company Novogel (Sfax, Tunisia), which is an agri-food company based in the new fishing port of Sfax and specialized in the preparation, freezing, and export of seafood (Figure 5).

Fresh crab samples (*n* = 50) were collected, and three different blue crab wastewaters were recovered from different industrial processing steps. All samples, placed in sterile bags or bottles, were put in a portable cooler for preservation during transportation to the lab.

Upon arrival at the laboratory and immediately under sterile conditions, seven blue crab-derived fractions and waters were prepared consisting of homogenized suspensions of flesh (FL), gills (GL), and viscera (VS), and carapace scraping water samples (CSW), as well as three crab wastewaters (WW1, WW2, and WW3). Each of the seven blue crab-derived samples (FL, GL, VS, SCW, WW1, WW2, and WW3) was prepared in triplicate.

To obtain homogenized suspensions of FL, GL, and VS, each 25 g portion of flesh, gills, or viscera, respectively, was stomached for 2 min with 100 mL of sterile physiological saline solution (TS solution composed of 0.85% NaCl, 0.1% peptone, and 1% Tween 80). For these fractions, almost two crabs were used for each of the three replicates. The CSW was obtained by simply scraping the shells of 7 randomly selected crabs with 30 mL of TS solution. In addition, the three collected wastewater samples (WW1, WW2, and WW3) were left to settle down and each upper part was recovered.

Finally, a total of twenty-one stock solutions were obtained corresponding to 7 crab-derived fractions with 3 replicates. For each sample, ten aliquots of 2 mL were frozen at −80 °C for conservation.

### 4.2. Enumeration of Bacterial Groups

Several appropriate serial 10-fold dilutions of each prepared stock solution were carried out in sterile physiological saline solution and 0.1 mL of each was spread-plated. Different bacterial populations were enumerated with appropriate media. Total psychrotrophic viable counts (TPVC) were determined using Long and Hammer agar (LH) supplemented with 1% NaCl [67] and incubated at 15 °C for 7 days. Halophilic heterotrophic flora was isolated on Zobell medium composed of aquarium salts (33.3 g L^−1^), yeast extract (1 g L^−1^), and tryptone (4 g L^−1^) and incubated at 27 °C for 3 days. Total lactic acid bacteria (LAB) were numbered on Elliker agar (Biokar Diagnostic, Beauvais, France) at 20 °C for 3 days under anaerobic conditions (Anaerocult A; Merck, Darmstadt, Germany). *Brochothrix* spp. were enumerated on streptomycin sulphate thallous acetate agar (STAA, Oxoid, Basingstoke, England) for 3 days at 20 °C. *Enterobacteriaceae* were quantified on a pour plate of violet red bile glucose agar (VRBGA, Biokar) incubated for 2 days at 30 °C. Bacterial concentrations were expressed as the logarithm (Log) of colony-forming units (CFU) per gram (CFU g^−1^) of crab fraction and Log (CFU mL^−1^) for water samples. Each type of sample was enumerated in triplicate.

### 4.3. Collection and Identification of Bacterial Isolates

#### 4.3.1. Bacterial Strain Collection

After plate counting analysis, bacterial strains were isolated from the countable Petri dishes. In addition to bacterial group enumeration, supplementary heterotrophic strains were also isolated from the BC samples on BHI-S (brain heart infusion agar (BHI, Biokar Diagnostics) with 2% NaCl), and plate count agar (PCA, Biokar Diagnostics) and were included in this collection.

The number of isolated bacterial strains were as follows: 33 isolates from GL, 30 from FL, 33 from VS, 31 from CSW, and 35, 40, and 45 from WW1, WW2, and WW3, respectively. Each strain was randomly selected by picking colonies from plates according to their different morphologies (color, form, and aspect). These 247 strains were isolated from LH (*n* = 66), Elliker (*n* = 58), BHI+ 2% NaCl (BHI-S) (*n* = 20), PCA (*n* = 19), and Zobell (*n* = 84). Each colony from LH, BHI-S, PCA, and Elliker was placed in 200 µL BHI; each colony isolated on Zobell was cultivated in 200 µL Zobell, respectively. The whole collection was built in 96-well microplates through incubation at 20 °C with shaking (150 rpm) to allow for subsequent medium-throughput identification and screening of EPS production. In parallel, cryotubes were also prepared for storage of cultures at −80 °C with 15% glycerol.

#### 4.3.2. Bacterial Isolate Identification by 16S rRNA Gene Sequencing

From the collection microplates, 50 μL culture broth was taken and placed in tubes containing 800 μL Zobell medium, then incubated for 24 h at 20 °C. A total of 100 µL of each culture was transferred to microplates with conical wells and centrifuged for 15 min at 4000 rpm and 4 °C. Supernatants were withdrawn and each pellet was dissolved in 100 μL sterile distilled water and heated for 5 min at 95 °C.

PCR was used to amplify 16S rDNA with the universal primers 8F (5′-AGAGTTTGATCATGGCTCAG-3′) and 1489R (5′-GTTACCTTGTTACGACTTCAC-3′), resulting in 1480 bp long amplicons. For the DNA polymerase, DreamTaq ready-to-use solution was used as a 2-fold master mix (Thermo Fisher Scientific, Waltham, MA, USA). In the PCR reaction mixture (23.5 µL), 1 µL of each primer (10 µM) and 1 µL of template DNA were added. PCR amplification was performed with My Cycler Thermocycler (Bio-Rad Laboratories, Marnes-La-Coquette, France) using the following protocol: initial denaturation (94 °C for 5 min), followed by 29 cycles of three steps including denaturation (94 °C for 30 s), primer annealing (55 °C for 30 s), and elongation (72 °C for 2 min). A final extension at 72 °C for 7 min was performed. PCR products were checked in a 1% (*w*/*v*) agarose gel containing Sybr Safe (Invitrogen, Waltham, MA, USA) and were subsequently pictured under UV illumination with Gel Doc XR+ imaging system and ImageLab software Version 3.0 (Bio-Rad Laboratories, Marnes-La-Coquette, France). PCR products were then purified using a GeneJet purification kit (Ref K0702, Thermo Fisher Scientific, Waltham, MA, USA) and sent for sequencing by Eurofins company (Köln, Germany).

Sequences were cleaned using Geneious Prime 2022.1.1 software (Biomatters, Auckland, New Zealand); the resulting sequences were then submitted to the Basic Local Alignment Search Tool program (BLAST) available at the National Center for Biotechnology Information (NCBI, Bethesda, MD, USA, http://www.ncbi.nlm.nih.gov/) for identification by similarity searches in the nucleotide collection (nr/nt). Taxonomy was investigated with the NCBI website accessed on 30 April 2023.

### 4.4. Blue Crab Microbiota 16S rDNA Gene Metabarcoding Analysis

#### 4.4.1. DNA Extraction

To separate bacterial cells from the crab matrix, 2 mL of the stock solutions was first centrifuged for 5 min at 400× *g* at room temperature. The pellet was discarded and the supernatant transferred into a new tube was centrifuged for 10 min at 13,000× *g*. The supernatant was removed and the bacterial cell pellet was used for DNA extraction following the DNeasy PowerFood Microbial kit procedure (Qiagen, Hilden, Germany.), with slight modification of the standard procedure as previously described by Jérôme et al. [68].

#### 4.4.2. Metabarcoding Analysis

Extracted DNA samples were sent to Eurofins company (Konstanz, Germany). Library preparation was performed by the company, targeting the V3-V4 region of the 16S rRNA gene using the primers 357F (5′-TACGGGAGGCAGCAG-3′) [69] and 800R (5′-CCAGGGTATCTAATCC-3′) [70] for 16S rRNA amplicon sequencing on the Illumina platform. The resulting sequences were analyzed using SAMBA (Standardized and Automated MetaBarcoding Analyses workflow) (https://github.com/ifremer-bioinformatics/samba, accessed on 21 September 2022). SAMBA is a FAIR scalable workflow integrating a unique tool verification of the integrity of raw reads and metadata (homemade script), as well as bioinformatics processing (QIIME 2 [71] and DADA2 [72]); it also consists of steps based on dbOTU3 [73] and microDecon to build high-quality amplicon sequence variant (ASV) count tables [74]. The SILVA 138.1 SSU Ref NR 99 database [75] was used to assign taxonomy to the ASVs using a naïve Bayesian classification. Alpha diversity was determined by species richness and Shannon diversity. The beta diversity was calculated with the Bray–Curtis dissimilarity index. Raw sequenced data are available at [76].

### 4.5. Screening for EPS-Producing Bacteria

#### 4.5.1. EPS Production Screening

Isolated strains were screened for EPS production. The inoculum (20 µL) was seeded in 1.2 mL transfer tubes containing 1 mL of 30 g L^−1^ glucose-supplemented medium, then incubated at 28 °C and 150 rpm for 48 h. These standard conditions were applied to all of the strains without optimization during this screening step [26]. The culture broth was centrifuged for 15 min at 4000 rpm. The supernatant was recovered and concentrated by ultrafiltration with NucleoFast 96 PCR plate for PCR clean-up (Macherey-Nagel, Hoerdt, France), using a vacuum pump.

#### 4.5.2. Agarose Gel Electrophoresis Analysis

Agarose gel (0.7% *w*/*v*) was prepared in TAE buffer (0.04 M Tris-acetate; 0.01 M EDTA, pH 8.5). A total of 30 mL of concentrated EPS samples was mixed with 10 µL of electrophoresis sample buffer (0.8 mL 10× TAE, 1.6 mL of glycerol, 5.2 mL of distilled water, 80 μL 0.5 M EDTA (5 mM final concentration), and 0.4 mL 0.5% bromophenol blue) and loaded into gel wells. Migration was performed at 150 V for 2 h with the TAE buffer using a refrigerated system. After migration, the gel was fixed with 25% (*v*/*v*) isopropanol for 1 h minimum and stained for 4 h or overnight in the dark with Stains-all (3,3′-Diethyl-9-methyl-4,5,4′,5′-dibenzothiacarbocyanine) solution prepared as follows: 5 mL Stains-all stock solution (0.1% *w*/*v* in dimethylformamide (DMF)), 5 mL DMF, 25 mL isopropanol, and 5 mL 300 mM Tris HCl pH 8.8; water was then added to 100 mL of the final solution. Gel was destained for 2 h in water under natural light, and finally analyzed with the GelDoc XR+ imaging system with ImageLab software Version 3.0 (BioRad, Marnes-la-Coquette, France).

### 4.6. EPS Characterization

#### 4.6.1. Preparation of the EPS

EPS production by bacterial strains was carried out in 600 mL of Zobell medium containing 30 g L^−1^ glucose in 2L Erlenmeyer baffled flasks incubated at 28 °C and 150 rpm for 48 h.

Centrifugation (45 min, 8000× *g* at 10 °C) was carried out and supernatants were filtered on 2.6 μm and 0.7 µm glass microfiber Whatman membranes (VWR International, Rosny-sous-Bois, France), then ultrafiltered using a 100 kDa cut-off membrane (Millipore, Fontenay-sous-Bois, France) and freeze-dried. Prior to analysis, the EPS was gently solubilized in water at 2 mg mL^−1^.

#### 4.6.2. Osidic Composition

Monosaccharide composition was determined according to the protocol described by Kamerling et al. [77] and modified by Montreuil et al. [78]. The EPS solution was firstly hydrolyzed for 4 h at 100 °C in methanol/HCl 3 N (Merck, Lyon, France). The obtained methyl glycosides were then converted to trimethylsilyl derivatives with the use of *N*,*O*-bis(trimethylsilyl)trifluoroacetamide and trimethylchlorosilane (BSTFA: TMCS) (Merck, Lyon, France) (99:1). Per-*O*-trimethylsilyl methyl glycosides formed were quantified using gas chromatography (GC-FID, Agilent Technologies, Les Ulis, France). The *myo*-inositol was used as the internal standard, as previously described [52].

#### 4.6.3. Molecular Weight Determination

The EPS weight-average molecular weight (Mw) was determined by size exclusion chromatography (SEC) (HPSEC Prominence Shimadzu Co., Kyoto, Japan) coupled with multiangle light scattering (MALS, Dawn Heleos-II, Wyatt Technology, Santa Barbara, CA, USA) and differential refractive index (RI) (Optilab Wyatt technology, Santa Barbara, CA, USA) detectors. The sample was prepared at 2 mg mL^−1^ and 100 µL was eluted on an Aquagel -OH mixed column (Agilent) that separates in the range 6 × 10^6^–5 × 10^3^ g mol^−1^. The MW was calculated by ASTRA software 6.8 (Wyatt technology Santa Barbara, CA, USA) using a refractive index increment dn/dc of 0.145 mL g^−1^ which is characteristic of polysaccharides [52].

## 5. Conclusions

This study reported, for the first time, the EPS production potential of bacterial strains isolated from *Portunus segnis.* The results clearly indicate the richness and diversity of the microbiota of the Tunisian blue crab *Portunus segnis*; it is mainly dominated by *Psychrobacter* spp., Lactic acid bacteria, and *Vibrio* spp.

Less than 20% of these isolated strains have been shown to produce anionic EPSs; their osidic characterization revealed interesting compositions, showing the presence of neutral, acidic, or amino sugars depending on the EPS. Among the selected strains, further investigation will focus on the innovative *Buttiauxella* strain and its EPS rich in both neutral and acidic sugars to highlight its biotechnological potential as a new GAG mimetic compound for the health domain. Further work will consist of (i) the production of selected EPSs in a bioreactor to assess their biotechnological potential and (ii) the study of their biological activities especially for biomedical applications. Bacterial EPSs are very attractive for replacing polysaccharides from animal origin, such as GAG which can result in a high risk of unknown cross-species contamination. EPSs rich in uronic acids can share some biological properties with GAG and their production by fermentation presents substantial advantages over animal extraction technology.

## Figures and Tables

**Table 1:** Identification of the bacterial strains isolated from the 4 blue crab samples (GL, FL, VS, and CSW) and 3 wastewaters (WW1, WW2, and WW3) from different industrial processing steps. GL: gills, FL: flesh, VS: viscera, CSW: carapace scraping water, and WW: wastewater.

Bacterial Species	Type of Samples							Total Number
	GL	FL	VS	CSW	WW1	WW2	WW3	
*Aeromonas hydrophila/salmonicida*							1	1
*Aeromonas salmonicida*	1							1
*Aeromonas* sp.		1			1			2
*Aeromonas veronii*					2			2
*Brochothrix thermosphacta*			1					1
*Buttiauxella brennerae*			1					1
*Buttiauxella* sp.	1							1
*Carnobacterium divergens*					1			1
*Carnobacterium maltaromaticum*	1							1
*Enterobacter* sp.							1	1
*Enterococcus faecalis*				4		1		5
*Enterococcus* sp.				1	1			2
*Escherichia coli*			1					1
*Jeotgalibaca dankookensis*			1			1	2	4
*Jeotgalicoccus nanhaiensis*							1	1
*Jeotgalicoccus* sp.						2	2	4
*Lactococcus garviae*	1	2	2	4	7	4	4	24
*Leclercia adecarboxylata*							1	1
*Lelliottia* sp.						1		1
*Macrococcus caseolyticus*		4			1			5
*Macrococcus* sp.	2	1						3
*Morganella morganii*			1	1	1			3
*Planococcus citreus*			1				1	2
*Planococcus rifietoensis*	1			1				2
*Planococcus* sp.	1							1
*Proteus hauseri*						1	1	2
*Proteus mirabilis*	1							1
*Proteus vulgaris*	1		1					2
*Pseudomonas shahriarae*	1							1
*Psychrobacter alimentarius*			1					1
*Psychrobacter arenosus*			2					2
*Psychrobacter celer*	2	3	1	2	3	6	4	21
*Psychrobacter cibarius*	1							1
*Psychrobacter cryohalolentis*						1		1
*Psychrobacter faecalis*			2				2	4
*Psychrobacter faecalis/pulmonis*				1	1			2
*Psychrobacter fozii*			3					3
*Psychrobacter glacincola*	1	2		1	1	1		6
*Psychrobacter halodurans*	2							2
*Psychrobacter marincola*	1	1	2	1	2		2	9
*Psychrobacter marincola/submarinus*	1		1	1		2	1	6
*Psychrobacter maritimus*	1	2	1		1	1	1	7
*Psychrobacter pulmonis*			1		1		1	3
*Psychrobacter* sp.	3	3		5	1	6	5	23
*Psychrobacter submarinus*			1		1	1	1	4
*Raoultella terrigena*					1			1
*Salinicoccus jeotgali/salsiraiae*							2	2
*Salinicoccus salsiraiae*		1						1
*Salinicoccus* sp.				1		2		3
*Serratia grimesii*	1					1		2
*Serratia liquefaciens*	3	2		1			1	7
*Serratia* sp.					1		1	2
*Shewanella algae*				2				2
*Shewanella indica*		1	1					2
*Shewanella* sp.					1		1	2
*Shigella sonnei*			1					1
*Staphylococcus pasteuri*		1						1
*Staphylococcus* sp.		1					1	2
*Streptococcus parauberis*	1		1					2
*Vagococcus fluvialis*				1	2	1	1	5
*Vagococcus* sp.			1	2	3	1	1	8
*Vibrio alginolyticus*	4	1	2	2	2	7	4	22
*Vibrio diabolicus/neonatus*		1						1
*Vibrio furnissii*	1	3	3				1	8
*Vibrio parahaemolyticus*							1	1
**Total number of isolates per sample**	**33**	**30**	**33**	**31**	**35**	**40**	**45**	**247**

**Table 2:** EPS-producing strains selected for further investigation. Their origin and identification, EPS production level, and weight-average molecular weight (Mw) are shown.

Code Number	Bacterial Isolate	Origin	Identification	EPS Production (mg L^−1^)	Mw (g mol^−1^)
1	CB3665	Gills	*Buttiauxella* sp.	44.3	15 × 10^6^
2	CB3564	Gills	*Psychrobacter* sp.	33.6	7.4 × 10^6^
3	CB3530	WW2	*Psychrobacter marincola/submarinus*	10.5	13 × 10^6^
4	CB3484	Viscera	*Psychrobacter pulmonis*	20	9.7 × 10^6^
5	CB3468	Gills	*Psychrobacter* sp.	17.6	32 × 10^6^
6	CB3464	Gills	*Psychrobacter halodurans*	22.1	11 × 10^6^
7	CB3791	WW3	*Shewanella* sp.	22.8	62 × 10^6^
8	CB3781	WW2	*Vibrio alginolyticus*	55.5	17 × 10^6^
9	CB3764	CSW	*Shewanella algae*	89.6	16 × 10^6^
10	CB3755	Viscera	*Vibrio alginolyticus*	85.3	14 × 10^6^
11	CB3765	CSW	*Vibrio alginolyticus*	127.8	81 × 10^6^
12	CB3737	Gills	*Vibrio furnissii*	77	36 × 10^6^

## Data Availability

Ifremer has an institutional website to share its data and publications https://archimer.ifremer.fr/, accessed on 23 January 2024.

## Supplementary Materials

The following supporting information can be downloaded at: https://www.mdpi.com/article/10.3390/molecules29040774/s1, Supplementary Data I: bacterial isolates identification and EPS production; 2.
